# Local Attraction of Substrates and Co-Substrates Enhances Weak Acid and Base Transmembrane Transport

**DOI:** 10.3390/biom12121794

**Published:** 2022-11-30

**Authors:** Nathan Hugo Epalle, Eric Beitz

**Affiliations:** Pharmaceutical Institute, Christian-Albrechts-University of Kiel, 24118 Kiel, Germany

**Keywords:** metabolite, transport, proton, proton wire, aquaporin, formate-nitrite transporter, ammonium transporter, monocarboxylate transporter, basigin, carbonic anhydrase, interaction, fusion

## Abstract

The transmembrane transport of weak acid and base metabolites depends on the local pH conditions that affect the protonation status of the substrates and the availability of co-substrates, typically protons. Different protein designs ensure the attraction of substrates and co-substrates to the transporter entry sites. These include electrostatic surface charges on the transport proteins and complexation with seemingly transport-unrelated proteins that provide substrate and/or proton antenna, or enzymatically generate substrates in place. Such protein assemblies affect transport rates and directionality. The lipid membrane surface also collects and transfers protons. The complexity in the various systems enables adjustability and regulation in a given physiological or pathophysiological situation. This review describes experimentally shown principles in the attraction and facilitation of weak acid and base transport substrates, including monocarboxylates, ammonium, bicarbonate, and arsenite, plus protons as a co-substrate.

## 1. Introduction

Transport of weak acid and base metabolites across the cell membrane is critical for numerous vital processes, including energy metabolism and pH regulation. Acidic metabolites, e.g., lactic, acetic, or pyruvic acid, exhibit pK_a_ values around 4, rendering them >99% deprotonated to their anionic form, i.e., lactate, acetate, and pyruvate, at neutral pH. Basic metabolites, e.g., ammonia, in turn, with pK_a_ values around 9, accept a proton under physiological pH conditions, giving rise to positively charged ammonium. As charged entities, the passage of such metabolites across cell membranes is strongly hampered.

Transport proteins facilitate the transfer of metabolite ions across the membrane by dealing properly with the accompanying protons. Contrary to primary active transporters that use the release of chemical energy from hydrolysis of ATP to transport even against existing transmembrane gradients, secondary active transporters, e.g., for lactate/H^+^, use the ionic force derived from the transmembrane gradient of one substrate to transport another. Their activity depends on the complex regulation of substrate and proton gradients around their transport sites.

Calculations indicate that the high cytosolic concentration in the millimolar range of household metabolites, such as lactate, pyruvate, and also ATP, make it impossible for the relatively slow transporters to deplete the concentration around their transport site before being regenerated by the Brownian diffusion [1]. For these high-concentration metabolites, the cytosol is comparable to a well-mixed compartment of homogenous concentration. However, the same is not true for the co-transported protons. Their much lower concentration in the nanomolar range seems at odds with the observed turnover rate of some transporters (85 s^−1^ for human monocarboxylate transporter 1 (MCT1)) [2]. The transporter activity should have depleted the substrate concentration around the entry sites, even taking into account that protons move five–seven times faster by the Grotthuss mechanism than diffusing ions. This suggests that weak acid metabolite transporters replenish the local concentration of their substrate and protons faster than simple diffusion would allow for [3]. In fact, micro-domains have been shown to exist at the transporting proteins themselves or at accessory proteins that locally increase substrate ion and/or proton concentrations for steeper transmembrane gradients. This occurs by attracting substrate molecules to the transporter entry sites, or by generating them in place by linked enzyme moieties.

This review describes processes by which metabolite transporters involved in the facilitation of low-concentration substrates maintain their transport functionality by local substrate enrichment. Specific examples of transporter proteins are used to illustrate these principles.

## 2. Electrostatic Attraction and Neutralization of Substrate Ions by the Transport Protein

One mechanism used by transmembrane facilitator proteins to attract substrate ions is exposing oppositely charged amino acids in electrostatic surface patches.

### 2.1. Substrate Attraction by Lactic Acid-Facilitating Aquaporins

Aquaporins (AQP) are a large, ancient family of homotetrameric channel proteins for water and neutral-solute transmembrane facilitation [4,5]. Two constrictions in the channels are highly conserved across the AQPs. One, termed the selectivity filter, is located close to the extracellular or periplasmic side of each AQP protomer and is typically composed of aromatic amino acids around a positively charged arginine (ar/R). The other lies in the center of the protomer and is named after its Asn-Pro-Ala signature motifs, i.e., NPA region [6]. Two NPA motifs cap two short helices at their positive ends. These positively charged constrictions act concertedly to strictly exclude protons and other cations [7,8]. In addition to vital functions in the human water and salt homeostasis, or glycerol metabolism, additional roles, e.g., in the modulation of the immune system, have been identified, rendering them attractive drug targets even though inhibitor development is hampered by the tight space in the substrate transduction path [9].

**Figure 1:**
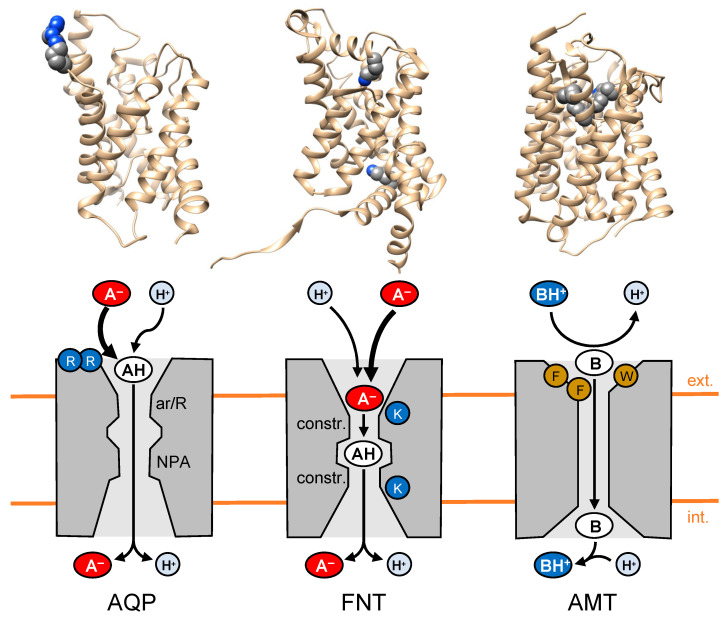
Schematic representation of models on the substrate binding, neutralization, and transduction of lactic acid-facilitating aquaporins, AQP (left; structure model), formate-nitrite transporters, FNT (center; PDB# 6vqq), and ammonium transporter/methylammonium permease/mammalian Rhesus proteins, AMT/MEP/Rh (right; PDB# 1u7g). Key amino acid residues are shown as spheres in the cartoons (backbone: sand; carbon: gray; nitrogen: blue).

Certain AQPs, e.g., from lactic acid bacteria [10] or human AQP9 [11], facilitate transmembrane diffusion at physiological pH conditions of lactic acid, as well as the typical AQP substrate spectrum [12]. The diffusion of lactic acid via such AQPs exceeds the buffer substrate concentration derived from the lactate/lactic acid protonation equilibrium (pK_a_ 3.86). Poisson–Boltzmann calculations of the electrostatic surface potential of respective AQPs revealed a strongly positively charged protein surface. To this end, the AQP9 tetramer, for instance, carries a cluster of eight arginine residues (4 × Arg51/Arg53). It was hypothesized that the positive surface charge attracts the predominant lactate anion form that indirectly enhances the local concentration of the neutral lactic acid substrate due to the protonation equilibrium (Figure 1, left) [11]. This view was supported by mutational replacement of the positive arginines by negatively charged glutamic acid residues. Indeed, the inversion of the AQP9 surface charge significantly decreased the passage of lactic acid.

The exclusive facilitation of neutral lactic acid via AQPs, and the subsequent dissociation into lactate/H^+^, can lead to a massive accumulation of lactate in the compartment at the less acidic side of the membrane, i.e., an ion trap [13]. In this compartment, protons are buffered, leaving the lactate ion that is excluded by the AQP and, thus, remaining trapped when there are no alternative transmembrane transporters with lactate-transport capability present.

### 2.2. The Next Step in Evolution: Channel-like Formate-Nitrite Transporters

Homopentameric formate-nitrite transporters (FNT) are expressed exclusively in microorganisms, mainly bacteria [14], but also in single-celled eucaryotes, such as malaria parasites [15]. Structure-wise, they almost perfectly mimic the fold of the AQP channel protomer, despite the absence of sequence similarity [16]. In terms of functionality, however, FNTs act like secondary-active transporters, using the transmembrane proton gradient as a driving force for the bi-directional transport of small, weak monoacids. As such, they are key elements in bacterial mixed acid fermentation [14], nitrogen fixation [17], and hydrosulfide detoxification [18]. The lactate/H^+^-transporting FNT from malaria parasites represents a novel, valid drug target [19,20] for which recently potent small-molecule inhibitors with high antimalarial potency have been discovered [21,22,23]. Similar to the AQPs, the substrate path through the FNT protein structure holds a central region that is flanked by two lipophilic constrictions and excludes the passage of charged compounds [17,24]. Nevertheless, weak acid substrate-transport is highly efficient even in the neutral pH range, indicating that the FNTs accept the anionic species as a substrate and make use of protons as a co-substrate [25]. How is this achieved?

The responsible feature in the FNT structure is the placement of a positively charged lysine each, deep inside two vestibule regions that lead to the lipophilic constrictions from either side of the membrane. Other than lactic acid-facilitating AQPs with a positive amino-acid cluster on the external protein surface, the FNTs steer the weak acid anion by charge attraction into an increasingly lipophilic protein environment. As a consequence, at a certain point along the pathway, the decreasing permittivity of the dielectric environment decreases the acidity of the substrate, leading to substrate protonation from the bulk and allowing the neutralized weak acid to cross the constrictions (Figure 1, center) [26]. We termed this mechanism the “dielectric slide” [27].

**Figure 2:**
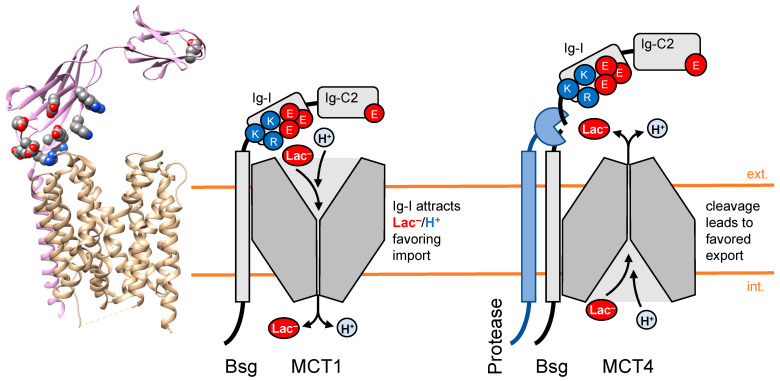
Contribution of the extracellular basigin (Bsg) Ig-domain to secondary-active MCT lactate/H^+^ transport (structure PDB# 6lz0). Two oppositely charged domains of the IgI domain act as a bivalent antenna for monocarboxylate substrate anions and co-substrate protons, increasing inward transport velocity and capacity (left schematic). Cleavage of the extracellular basigin domain by a protease promotes lactate/H^+^ export via MCT4 (right). Key amino acid residues are shown as spheres in the cartoon (Bsg: pink; MCT1: sand; carbon: gray; nitrogen: blue; oxygen: red).

As non-flexible membrane proteins with an internal rigid and narrow substrate pathway, the FNTs are clearly channel-like. Furthermore, the entry sites on both sides of the membrane are permanently accessible to substrates. Such properties contradict the classical definition of transport proteins, according to which a substrate is bound only at one open side, the cis side, followed by a large conformational change of the protein that opens up the trans side for substrate release (secondary-active transporters are discussed in Section 3 and depicted in Figure 2). However, the FNT transport activity is equally efficient as that of classical secondary-active monocarboxylate transporters, showing that the FNT class of proteins represents a linking intermediate between channels and transporters.

### 2.3. Weak Base Transport: Opposite Prerequisites and Requirements

Weak bases, such as ammonia (NH_3_) accept a proton at physiological pH, giving rise to positively charged ammonium (NH_4_^+^) which represents the predominant species. Transmembrane facilitators for ammonium are present and conserved across all domains of life, named the ammonium transporter/methylammonium permease/mammalian Rhesus protein family (AMT/MEP/Rh) [28]. The proteins assemble as homotrimers at the level of the cell membranes, with each protomer carrying a pore capable of passing neutral ammonia; however, the protonation status of the substrate still appears to be debated. The situation in terms of protein structures and substrate charge neutralization is highly reminiscent of that discussed above for the AQPs and FNTs. However, contrary to weak acid anions, in order to convert a cationic weak-base substrate into a neutral molecule, it needs to release a proton rather than accept one.

A well-accepted model states that NH_4_^+^ recruitment and deprotonation is achieved by AMT/MEP/Rh proteins via a triad of aromatic amino acids, e.g., Phe107, Trp148, Phe215 in bacterial AmtB, in the periplasmatic vestibule (Figure 1, right) [29]. The electron-rich aromatic environment facilitates NH_4_^+^ binding by cation–pi interactions, and, at the same, the lipophilicity of the residues promotes deprotonation of ammonium to form neutral NH_3_. The neutral NH_3_ is compatible with the hydrophobic interior of the transduction path. After release into the cytosol, ammonia will be immediately re-protonated (Figure 1). Experimental studies on ammonia/ammonium transport are hampered by technical challenges related to the small size, background membrane diffusion, and the interconvertibility of the substrate protonation species, depending on the pH situation on either side of the membrane. Therefore, conflicting reports are present in the literature, showing, for instance, that proposed key residues—including the Phe107/Trp214/Phe215 triad [30] and two conserved histidines in the center of the transport path [31]—could be mutated without a loss of transport functionality, arguing that these amino acids are not essential. Details of such debates are summarized in another review [32].

## 3. Chaperones of Transport Proteins Act as Local Attractors for Substrates

Chaperone proteins appear to be involved in additional processes besides their classical functions in the control of protein quality and folding, or the intracellular trafficking of proteins to their proper cellular location. In this sense, the chaperone basigin of secondary-active mammalian monocarboxylate transporters (MCT) has been identified to provide an extracellular bivalent-collecting antenna for the substrate (mainly lactate) and the proton co-substrate, shifting the directionality of transport inwards.

The MCT class of membrane proteins belongs to the solute carrier SLC16A family. It shuttles lactate, pyruvate, and acidic ketone bodies across the cell membrane [33]. The MCT1 and MCT4 isoforms are at the center of the mutually beneficial lactate transport between the hypoxic, i.e., glycolytic, and the oxidative, i.e., lactate-consuming, cancer cells (Warburg and reverse-Warburg effects) [34], as well as in the lactate shuttle between glycolytic astrocytes and oxidative neurons in the brain [35]. Consequently, MCT inhibitors are in clinical development for the treatment of certain types of cancer [36]. The trafficking of MCT1 and MCT4 from the Golgi compartments to the cell membranes depends on the presence of the chaperone protein, basigin (CD147) [37,38]. Basigin, and a second, MCT2-associated chaperone, embigin [39], harbor a single transmembrane domain as a membrane anchor and interaction site with the MCT [36]. The intracellular C-terminus of basigin is short, whereas the extracellular part is composed of repeated immunoglobulin-like (Ig) domains. The ubiquitously expressed splice-variant 2 of basigin harbors two Ig-like domains named Ig-I and Ig-C2 [40]. The retina-specific variant 1 carries a third Ig-0 domain [41], and two shorter variants, 3 and 4, exist with only the membrane-proximal Ig-I domain present.

A recently generated cryo-electron microscopy structure of the MCT1-basigin complex shows that the Ig domains form a slightly open lid-like structure, forming a micro-compartment above the extracellular transporter entry site (Figure 2, left) [36]. MCT1 protein structures were obtained in the presence of small-molecule MCT inhibitors that locked the transporter either in the outward-open (inhibitors AZD3965 and BAY-80029) or the inward-open conformation (7ACC2) [36]. By expression of basigin-MCT1 fusion proteins, we could show that the presence of the basigin Ig-I domain increased the achievable concentration of intracellular lactate, i.e., the transport capacity, by a factor of 4–5 [40]. We were able to assign this effect to two amino acid patches of opposite charge in the Ig-I domain, i.e., a negative patch consisting of Glu114, Glu118, Glu120, Glu168, and Glu172 next to a positive patch with Lys108, Lys111, Lys127, Arg201, and Arg208. Replacing the charged amino acid residues of either patch with neutral ones resulted in decreased transport [40]. In conclusion, the Ig-I domain of basigin appears to act as a bivalent antenna for lactate anions (positive surface patch) and for protons (negative patch). The resulting rise in the local concentrations of substrate and co-substrate close to the MCT1 entry site enables higher transport rates and increased transport capacity due to steeper transmembrane gradients then present in the bulk.

The contribution of the basigin Ig-I domain to MCT-facilitated lactate/H^+^ transport is of (patho-)physiological relevance, as shown by a study that identified a transmembrane protease that is expressed in human lung squamous cell carcinomas and cleaves off the extracellular domain of basigin (Figure 2, right) [42]. The authors found that removal of the basigin Ig domains shifts the directionality of MCT4-associated lactate transport by a factor of 4, increasing the malignancy of the tumor cells.

## 4. Carbonic Anhydrases Contribute Non-Catalytically to Proton-Driven Transport

Members of the carbonic anhydrase family (CA), especially the isomers CAII, CAIV, and CAIX, have been found to associate with a variety of transporters, including the MCTs. Due to their enzymatic function, i.e., reversible hydration of CO_2_ into bicarbonate and protons, CAs have physiological roles in the acid/base equilibrium of the cells, which is also of relevance in the context of cancer. Unexpectedly, unrelated to their catalytic enzyme properties, CAs were found to be involved in metabolite transport [43].

### 4.1. Extracellular CAIV

**Figure 3:**
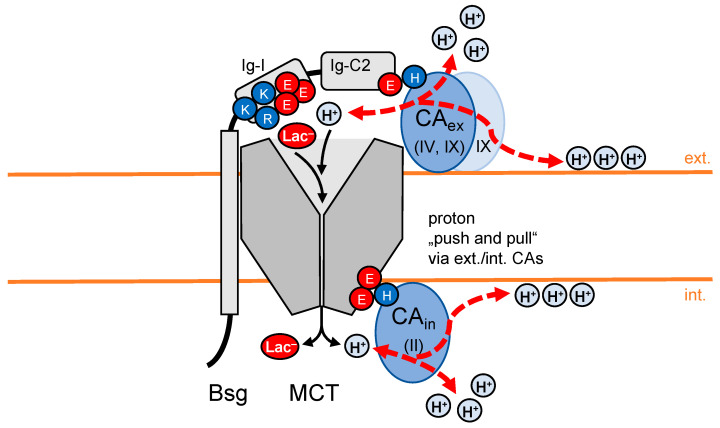
MCT transport supported by CA proton antennae. The extracellular isoforms CAIV or CAIX, and the intracellular CAII, contribute non-catalytically to MCT transport by channeling protons to and from the MCT entry sites according to a “push and pull principle”.

The extracellular isoform CAIV is fixed to the surface of the cells by a GPI anchor at its C-terminus (Figure 3) [44]. Additionally, it forms an electrostatic interaction via a positively charged histidine to a glutamate in the Ig-C2 domain of basigin [45], or to an aspartate/arginine ion pair of embigin [39,45]. The association of CAIV via embigin to MCT2 has been shown to increase the lactate transport. Neither application of the CA inhibitor 2-benzothiazolsulfonamid, EZA, nor impairment of the catalytic activity of CAIV by point mutation, diminished the positive effect on the MCT2 transport [39]. The authors concluded from the data that the CAIV contribution is non-catalytical but related to a proton antenna function. Related studies on CAIV in connection with MCT1 and MCT4 via basigin were in line with the CAIV-embigin-MCT2 system [46,47].

### 4.2. Extracellular CAIX

The extracellular isoform CAIX is mainly expressed in the stomach and intestine. Structural studies showed that the CAIX protein structure comprises a globular catalytic domain and a membrane-anchoring helix at the C-terminus. CAIX further assembles into homodimers (Figure 3). In the same way as CAIV, CAIX can form a complex with the chaperone basigin via interaction of a histidine and the before-mentioned glutamate in the Ig-C2 domain [48]. The effect of CAIX on associated basigin/MCT was measured on the cellular level by knockdown of CAIX expression in cancer cell lines, which decreased lactate transport. Again, the involvement of CAIX in MCT transport was shown to be non-catalytical, supporting the hypothesis of a proton-collecting antenna function [48,49]. Since the transcription of the CAIX gene is upregulated in hypoxic tumor cells by the hypoxia-induced transcription factor HIF-1α, CAIX may act as a malignancy factor by enhancing the release of lactate [50].

### 4.3. Intracellular CAII

The intracellular isoform CAII interacts directly with MCTs. It binds via a histidine to clusters of glutamate residues in the intracellular C-terminal region of the MCT1 [51] and MCT4 (Figure 3) [52]. However, it does not appear to interact with MCT2 [39]. As shown for the extracellular CAs, abolishment of the catalytic activity of CAII by inhibitors or point mutation maintained the positive effect on MCT-facilitated lactate transport [53,54]. Computational models [55] and confirming experiments using respective CAII point mutants established that two specific negatively charged amino acid residues, Glu69 and Asp72, are responsible for the supply of protons to the MCT or removal, respectively, depending on the current export or import directionality [53].

### 4.4. “Push and Pull Principle” of Fully CA-Decorated MCT

When both an extracellular and an intracellular CA concurrently interact with an MCT, the complex may act according to a “push and pull principle” [46]. The CA on the membrane side with the higher proton concentration “pushes” the collected protons from the bulk or membrane surface towards the transporter entry site, while the oppositely positioned CA will expedite the transport process by “pulling” from the other side (Figure 3). Such a setup would result in higher proton-driven lactate transport velocities than a system that solely depends on the diffusional provision of its substrates. Ordered pathway structures may further help in the dissipation of protons away from the transporter exit, providing connections with the membrane surface (see Section 6) or other proton acceptors for dispersion [56,57].

## 5. Transporter-Associated Enzymes Provide Substrates in Place, Enhancing Transport

The directionality of the chemical equilibrium reaction catalyzed by CAs depends on the substrate availability. This means CA activity can either generate or use bicarbonate and protons:

CO_2_ + H_2_O ⇆ HCO_3_^−^ + H^+^ (1)

Transporters that use bicarbonate or protons as substrates or co-substrates can, thus, be affected in their transport activity if a CA enzyme in the proximity creates a local enrichment of the respective molecules.

### 5.1. CA Activity Increases Activity of Proton-Driven Lactate Transport by Locally Generating Protons

Physiological studies suggested that not only the proton antenna function but also the enzymatic activity of CA contributes to MCT-facilitated lactate transport, e.g., in astrocytes [58] and skeletal muscles [59]. In these cases, the cytosolic acidification derived from lactate/H^+^ influx could be blocked by the inhibition of the extracellular CA activity, presumably extracellular CAIV, by more or less specific small-molecule inhibitors. Such data show the complexity of a system in which protons are generated, shuttled, and used as co-substrates by the various involved protein components, because direct proton transfers, and indirect pH effects or buffering by the bulk, intertwine and need to be resolved for specific assignment.

### 5.2. CA Activity Increases Activity of the Na^+^/H^+^ Exchanger by Locally Generating Protons

**Figure 4:**
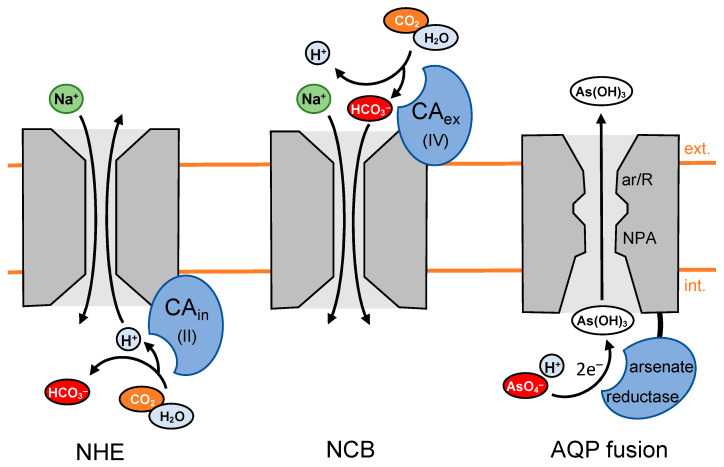
Enhancement of transmembrane transport by enzyme activity, providing weak acid/base substrates or co-substrate close to the transporter entry sites. Shown are co-localizations of CA isoforms with the sodium–proton exchanger, NHE (left), and the sodium-bicarbonate symporter, NCB (center), as well as a fusion protein between an aquaporin and an arsenate reductase (right).

The intracellular CAII has further been shown to interact with the C-terminus of the ubiquitous Na^+^/H^+^ exchanger, NHE [60,61], i.e., a transporter required for intracellular pH homeostasis. The interaction appears to increase the activity of the NHE, requiring catalytic activity of CAII in order to provide protons to the exchanger (Figure 4, left). Another report states that the NHE activity was also affected by the activities of extracellular CAIV [62] and CAIX [63]. The role of the extracellular CAs in the process was attributed to the swift dissipation of the protons at the exit site of the exchanger.

### 5.3. CA Activity Increases HCO_3_^−^ Transport by Locally Generating Bicarbonate

CA catalytic activity is also involved in the transmembrane transport of bicarbonate [64,65,66]. Specifically, the Na^+^/HCO_3_^−^ co-transporter, NCB, interacts with extracellular CAIV, which generates bicarbonate close to the transporter entry site (Figure 4, center). The provision of substrate increases transport [67]. A supportive role has been assigned to CAII located at the opposite, intracellular side of the membrane due to its enzymatic activity in the dissipation of bicarbonate at the exit site of the transporter [68,69].

In a similar fashion, the Cl^−^/HCO_3_^−^ exchangers, AE, were stated to benefit from the activity of CAIX by removing bicarbonate exported out of the cell from the antiporter exit site [70]. The intracellular CAII isoform possibly binds to AEs providing bicarbonate to the entry site [71,72,73]. However, several groups challenge the notion that CAII interacts directly with NCB or AE [74,75,76,77] or contributes to transport via NCB by generating bicarbonate [77]. Details of the matter are discussed elsewhere [64,65,78].

### 5.4. Channel-Enzyme Fusion Proteins Generate and Compartmentalize Substrates as a Single Entity

Fusions on the genetic level between transmembrane facilitators and catalytically active enzymes that generate the substrate in place are rare [79]. However, in situations where the swift release of a compound, e.g., a toxin, is beneficial or even vital for a cell, this concept enables confinement of the compound and extrusion in an energy-saving fashion by generating a steep local transmembrane gradient. In this sense, AQP channels of the aquaglyceroporin type from soil and marine bacteria, e.g., *Mycobacterium tuberculosis* or *Salinispora tropica,* that carry C-terminally fused small arsenate-reductase domains (Figure 4, right) have been identified and studied [80]. The bacteria cannot prevent the uptake from the environment of toxic arsenate ions, AsO_4_^−^, via phosphate transporters. For detoxification, arsenate will be reduced by arsenate reductase enzymes, forming arsenite, which is the anion of arsenous acid. Its very weak acidity (pK_a_ 9.2) leads to immediate protonation, generating neutral As(OH)_3_ under physiological pH conditions. As(OH)_3_, in turn, is structurally similar to glycerol and can pass the aquaglyceroporin-type channel domains of the fusion proteins. The simplicity of the substrate generation/extrusion mechanism by channel–enzyme fusions is striking. However, evolution apparently preferred more complex systems consisting of several interacting proteins, as previously described in this review, probably due to the higher degree of potential adjustability and regulation.

## 6. Lipids of the Cell Membranes Facilitate Proton-Coupled Transport

Transmembrane transport proteins are embedded in the lipidic environment of the cell membranes, and transport properties in terms of kinetics and selectivity of several membrane proteins have been shown to be modulated by the lipid composition. In the scope of this review, the polar membrane surfaces have been found to facilitate the collection and swift transfer of protons to associated membrane proteins (see Figure 3) [56]. Specifically, the more proximal lipids within a 30–60 nm^2^ area around proton-accepting proteins increase protonation events [81]. However, long-range proton transfers of about 100 nm were also measured, when proton gradients were generated along the membrane by transmembrane transporters acting as a sink [56].

Different proton-diffusion models were proposed to describe the experimental outcome [82,83]. Accordingly, the protons may bind directly to the polar lipid head groups and hop along [84], or they may diffuse in the layer of water molecules interfacing the bulk with the membrane lipids [85].

Together, the proton-collecting function of the two-dimensional membrane surface from the three-dimensional aqueous bulk and delivery to transport proteins further facilitates transmembrane proton transport and proton-coupled transport.

## 7. Conclusions

The transmembrane transport of weak acid and base metabolites depends on several interconnected factors, i.e., the availability of the actual substrate, the pH conditions that determine the charge of the substrate by protonation, the lipid environment, and the presence or enzymatic activity of associated proteins that directly shuttle protons or indirectly feedback on each level. This unfolding degree of intricacy is the product of evolving adaptations to the various situations a cell has to deal with in its individual physiological context. Such complexity should be appreciated and considered when studying the physiological implications of weak acid and base transport functionality. From a therapeutic point of view, these strong interdependences may further create more angles of attacks in the pursuit of target proteins for modulating transport activity.
